# Supplementary material for: Effect of Culture Supernatant of Clostridium butyricum TO-A on Human DNA-Repair-Factor-Encoding Gene Promoters
Source: Int J Mol Sci. 2024 Nov 12;25(22):12151. doi: 10.3390/ijms252212151 (PMC11594347; doi:10.3390/ijms252212151)
Supplement: Supplementary file 1 [file ijms-25-12151-s001.zip › Supplementary Materials/Supplementary Figure 1.pptx]

## Slide 1
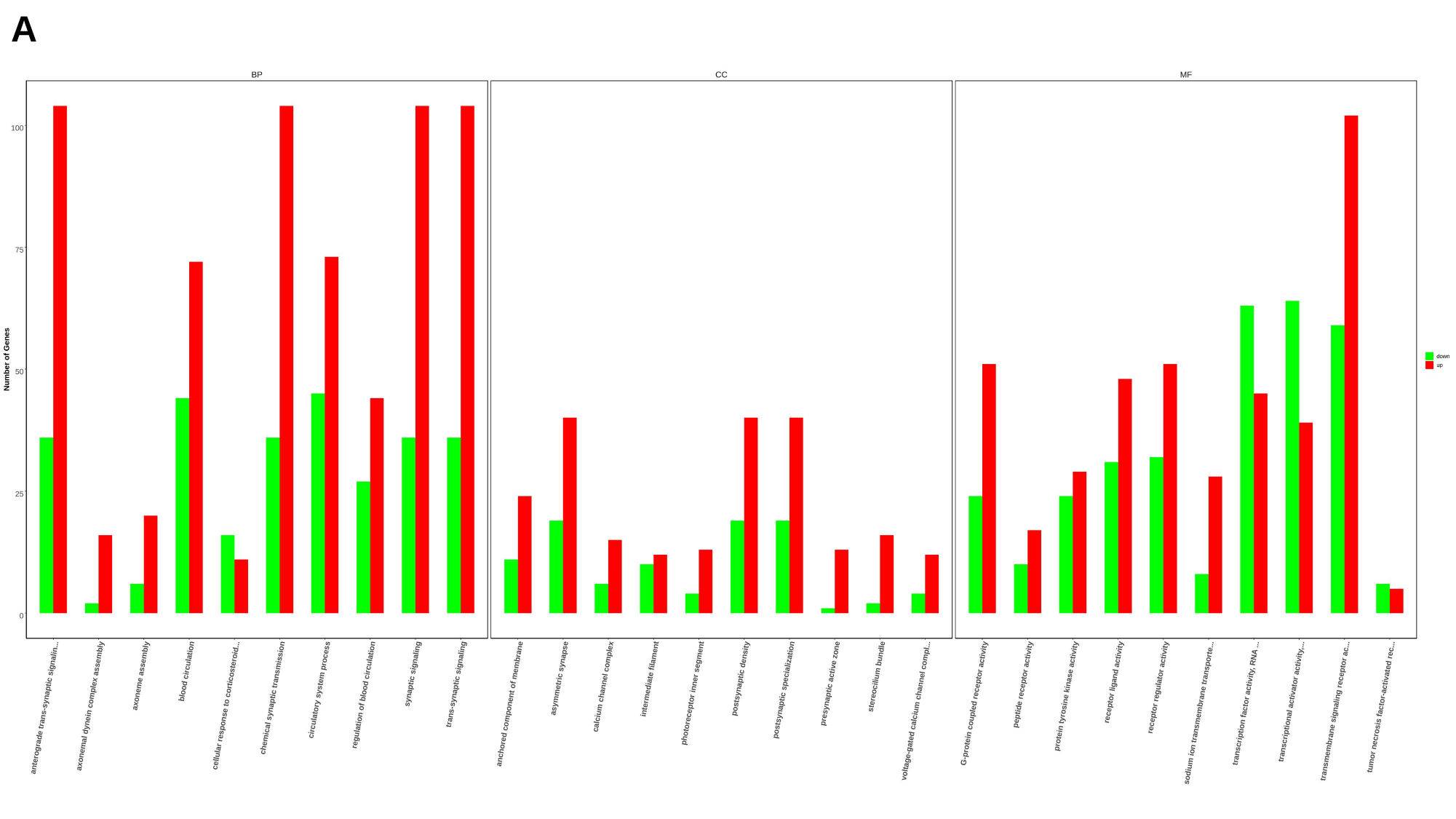

A

## Slide 2
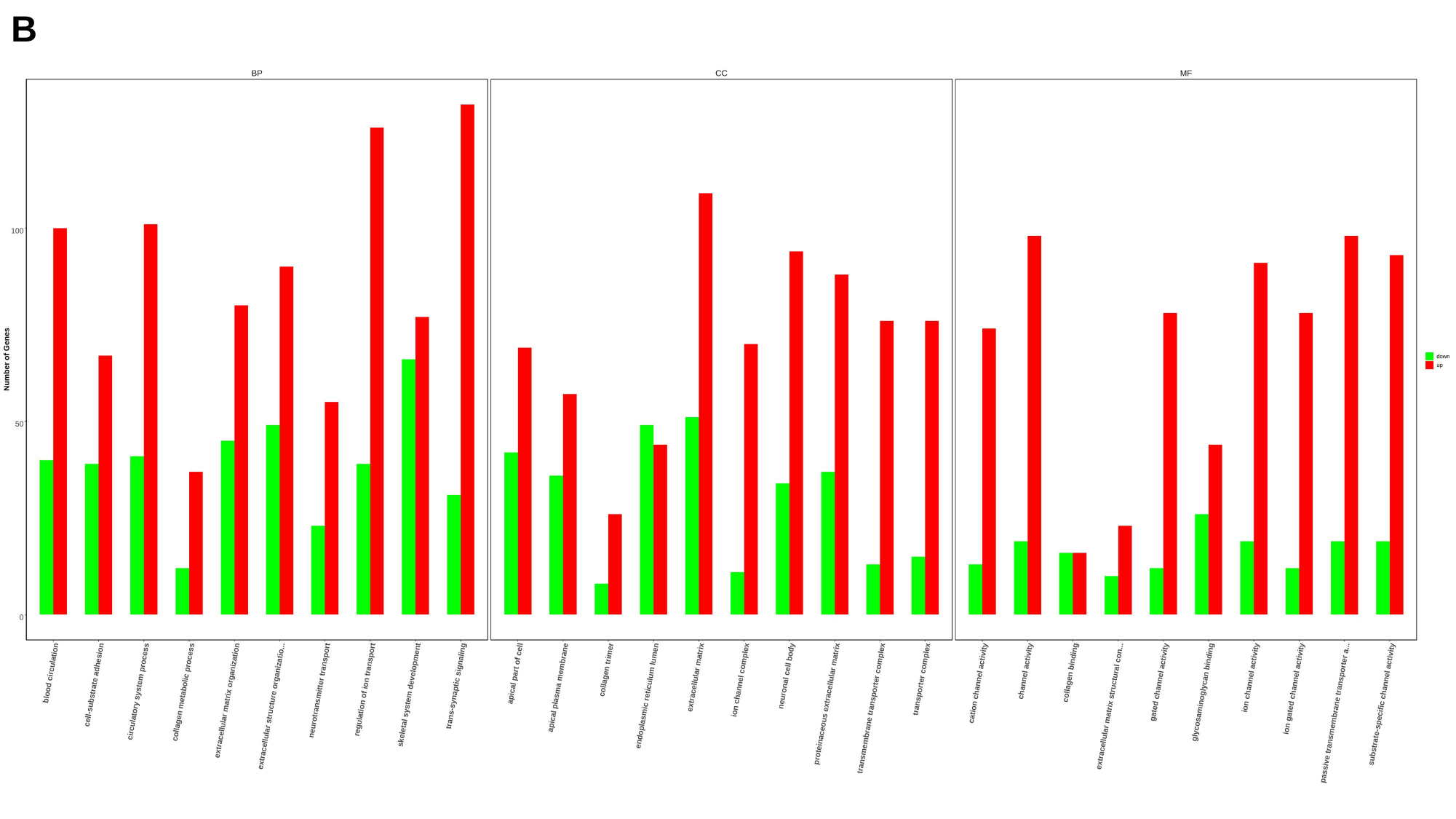

B

## Slide 3
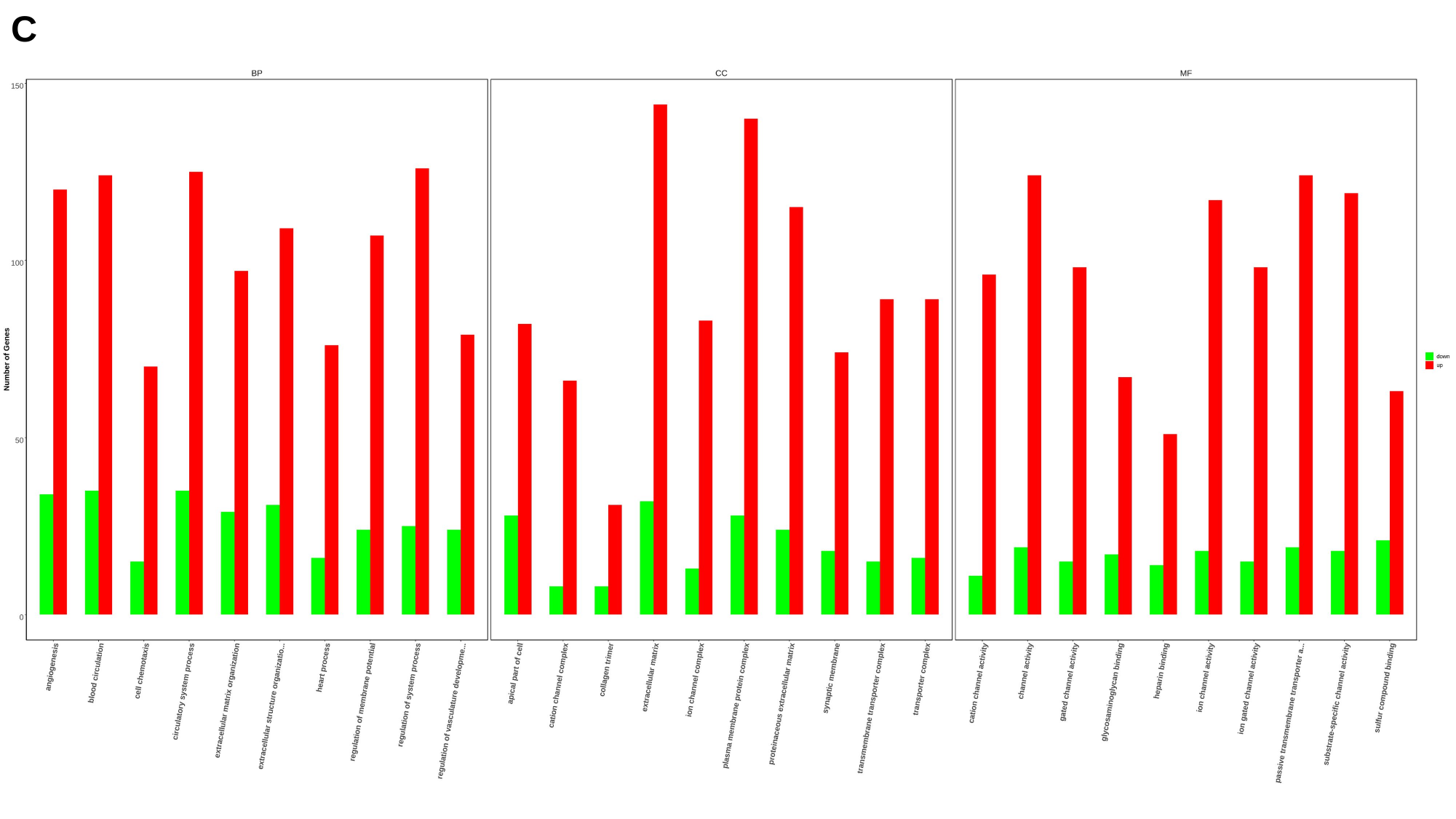

C

## Slide 4
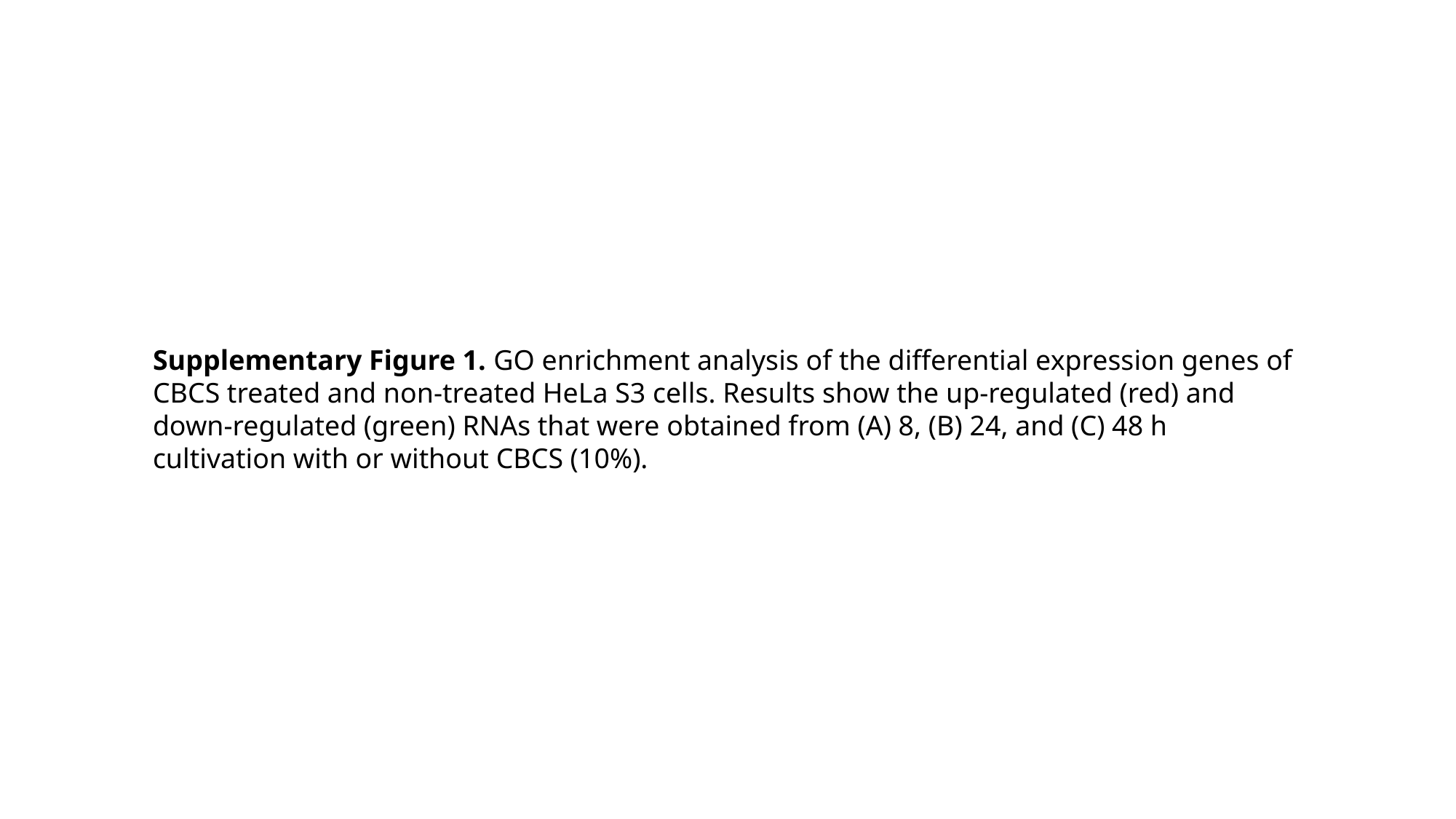

Supplementary Figure 1. GO enrichment analysis of the differential expression genes of CBCS treated and non-treated HeLa S3 cells. Results show the up-regulated (red) and down-regulated (green) RNAs that were obtained from (A) 8, (B) 24, and (C) 48 h cultivation with or without CBCS (10%).
